# *Toxoplasma gondii* seropositivity and serointensity and cognitive function in adults

**DOI:** 10.1371/journal.pntd.0008733

**Published:** 2020-10-15

**Authors:** Shawn D. Gale, Lance D. Erickson, Evan L. Thacker, Elizabeth L. Mitchell, Bruce L. Brown, Dawson W. Hedges

**Affiliations:** 1 Department of Psychology, Brigham Young University, Provo, Utah; 2 The Neuroscience Center, Brigham Young University, Provo, Utah; 3 Department of Sociology, Brigham Young University, Provo, Utah; 4 Department of Public Health, Brigham Young University, Provo, Utah; 5 Department of Statistics, Brigham Young University, Provo, Utah; Centre hospitalier de Cayenne, FRANCE

## Abstract

Infecting approximately one-third of the world’s human population, *Toxoplasma gondii* has been associated with cognitive function. Here, we sought to further characterize the association between *Toxoplasma gondii* and cognitive function in a community sample of adults aged approximately 40 to70 years. Using adjusted linear regression models, we found associations of *Toxoplasma gondii* seropositivity with worse reasoning (b = -.192, *p* < .05) and matrix pattern completion (b = -.681, *p* < .01), of higher anti-*Toxoplasma gondii* p22 antibody levels with worse reasoning (b = -.078, *p* < .01) and slower Trails (numeric) performance (b = 5.962, *p* < .05), of higher anti-*Toxoplasma gondii* sag1 levels with worse reasoning (b = -.081, *p* < .05) and worse matrix pattern completion (b = -.217, *p* < .05), and of higher mean of the anti-*Toxoplasma gondii* p22 and sag1 levels with worse reasoning (b = -.112, *p* < .05), slower Trails (numeric) performance (b = 9.195, *p* < .05), and worse matrix pattern completion (b = -.245, *p* < .05). Neither age nor educational attainment moderated associations between the measures of *Toxoplasma gondii* seropositivity or serointensity. Sex, however, moderated the association between the sag1 titer and digit-symbol substitution and the association between the mean of the p22 and sag1 levels and digit-symbol substitution, and income moderated the association between *Toxoplasma gondii* seropositivity and numeric memory and the association between the p22 level and symbol-digit substitution. Based on the available neuropsychological tasks in this study, *Toxoplasma gondii* seropositivity and serointensity were associated with some aspects of poorer executive function in adults.

## Author summary

*Toxoplasma gondii* is a parasite that reproduces in cats but that can infect other animals and humans. *Toxoplasma gondii* has an affinity for the brain and infection results in a long-term immune response that may have negative effects. Animal models have shown that *Toxoplasma gondii* can affect behavior and cognition (thinking). In humans, the association is less clear, but there are associations between infection and variety of psychiatric and neurological conditions such as schizophrenia and epilepsy. *Toxoplasma gondii* may even increase the risk of dementia. If true, it is possible that the presence of this infection may affect thinking long before the onset of dementia. We sought to determine if the presence of antibodies in the blood to Toxoplasma gondii, which is the immune system’s response to past infections, might be associated with performance on measures of cognition. We analyzed a large dataset, the UK Biobank, which includes measures of cognition and blood test results for antibodies to *Toxoplasma gondii*. We found that both the presence of antibodies as well as the amount of these antibodies were related to some aspects of cognition, particularly problem solving and reasoning in otherwise healthy adults ages 40 to 69 years.

## Introduction

Accumulating evidence indicates that some infectious diseases might be associated with cognitive function and dementia [1–3]. For example, meta-analyses have shown associations between *Chlamydia pneumoniae* and dementia and between spirochete infection and dementia [4], and a systematic umbrella review found suggestive evidence of an association between herpesviridae viruses and Alzheimer’s disease [5], findings that together suggest the importance of considering the associations between microbial pathogens and both cognitive decline and dementia [3].

Emerging evidence also suggests a possible association between the apicomplexan protozoan *Toxoplasma gondii* (*T*. *gondii*) and cognitive function and dementia. Infecting approximately one-third of the world’s human population [6] and having a worldwide distribution [7], *T*. *gondii* can persist in the brain for the life of the host [8]. In addition to possibly influencing human behavior [8–10] and showing an association with schizophrenia [11], some [12–18] but not all [19–21] evidence suggests that *T*. *gondii* in humans is adversely associated with cognitive function. Moreover, the results of a recent meta-analysis showed an association between *T*. *gondii* and dementia [22].

The differences in previous findings have resulted in an incomplete characterization of the association between *T*. *gondii* and cognitive function in humans. To contribute to this emerging literature, we sought to characterize further the association between *T*. *gondii* and cognitive function in adults using the UK Biobank, a large community-based sample that has data about exposure to *T*. *gondii*, a battery of tasks assessing executive function and memory, and a range of demographic and medical variables by which to control for possible cofounding.

## Methods

### Study sample

The sample for this study is a subset of participants in the UK Biobank Resource, a large community-based sample of adults. The UK Biobank received ethical approval from the National Research Ethics Service Committee North West-Haydock (reference 11/NW/0382). All participants gave consent (http://biobank.ctsu.ox.ac.uk/crystal/field.cgi?id=200). We received approval to use anonymized data from the UK Biobank under application number 41535. Between 2006 and 2010, the UK Biobank enrolled approximately 500,000 adults with the majority aged 40 to 69 years. Participants were sampled from population-based registries (http://www.ukbiobank.ac.uk) and accessed at 22 centers across the United Kingdom [23]. UK Biobank data collection included biological samples, nurse interviews, physical examinations, and questionnaires to obtain demographic and medical information from participants (http://biobank.ctsu.ox.ac.uk/crystal/field.cgi?id=200). Individuals who contributed data to the UK Biobank are not representative of the general population (and hence cannot be used to provide representative disease prevalence and incidence rates). Despite not being representative of the general population, findings from the UK Biobank dataset can still provide valid estimates of associations between exposure and disease (http://www.ukbiobank.ac.uk/wp-content/uploads/2017/03/access-matters-representativeness-1.pdf)

Our analyses are limited to the intersection of participants who had valid data for both *T*. *gondii* (https://biobank.ctsu.ox.ac.uk/crystal/crystal/docs/infdisease.pdf) and the various cognitive functioning measures. There were 9,341 participants who had *T*. *gondii* data. Participants who were given cognitive functioning tests were administered different combinations of the tests; therefore, there is a range of sample sizes for the various analyses. Missing data due to abandoned tests was quite small. Because some of the tests considered abandoning the effort as a form of data, some tests had no missing data due to non-response, and the maximum of missing data because tests were not completed was three percent. In other words, missing data on the cognitive functioning tests was predominantly by design. We then excluded participants with missing data on other model covariates. Samples for analyses therefore ranged from 301 to 6,780 (Table 1).

**Table 1:** Descriptive statistics of study variables.

	Mean	SD	Minimum	Maximum	N
Cognition					
Numeric memory	6.77	1.31	2	12	795
Reasoning	6.37	2.09	0	13	2,267
Pairs matching: Incorrect	4.10	3.35	0	39	6,780
Matrix pattern completion	8.36	1.99	2	14	312
Tower rearrangement	10.49	3.59	0	18	316
Symbol-digit substitution	19.78	4.94	8	36	313
Reaction time	547.65	108.48	320	1594	6,752
Trails: Numeric	214.68	71.11	107	692	312
Trails: Alphanumeric	542.04	244.99	244	2442	301
*Toxoplasma gondii*					
*T*. *gondii* seropositive	.27		0	1	6,780
ln(p22)^a^	3.39	1.34	0	9	6,780
ln(sag1)^a^	4.43	.95	0	9	6,780
Mean of ln(p22) and ln(sag1)^a^^,^^b^	-.02	.90	-4	3	6,780
Age	55.30	8.13	40	70	6,780
Female	.55		0	1	6,780
White	.95		0	1	6,780
College degree	.41		0	1	6,780
Income (in 10,000 £)	4.46	3.10	1	12	6,780
Self-rated health	2.93	.73	1	4	6,780
Body-mass index	27.16	4.73	16	61	6,780
Smoking status					
Non-smoker	.58		0	1	6,780
Past	.33		0	1	6,780
Current	.09		0	1	6,780
Drinking frequency					
Daily or almost daily	.23		0	1	6,780
3–4 times/week	.24		0	1	6,780
Once or twice/week	.25		0	1	6,780
1–3 times/month	.11		0	1	6,780
Special occasions	.11		0	1	6,780
Never	.06		0	1	6,780

Note

^a^ Antibody serointensity in median florescence intensity.

^b^ ln(p22) and ln(sag1) were standardized before being averaged. Source: *UK Biobank*.

### Toxoplasma gondii

*T*. *gondii* infection was determined based on p22 and sag1 antigen levels in units of median florescence intensity [24]. Participants were considered to be *T*. *gondii* seropositive if antibody levels to the p22 antigen were greater than 100 or if antibody levels to the sag1 antigen were greater than 160 (https://biobank.ctsu.ox.ac.uk/crystal/field.cgi?id=23062). The methodology related to these cutoffs for determining seropositivity is described elsewhere [24]. In some estimated models, we used serointensity of p22 and sag1, both natural-log transformed, and the mean of the natural-log transformed versions of p22 and sag1 after standardization (i.e., we mean centered and divided each natural-log transformed version by its standard deviation) as the independent variable.

### Assessment of cognitive function

In the UK Biobank, cognitive function was assessed with a battery of neuropsychological tasks. Numeric memory assesses working memory by recall of increasing numbers of digits (higher score is better). Reasoning assesses fluid intelligence by correct answers to problem-solving questions that require logic and reasoning (higher score is better). Pairs matching requires participants to recall the position of matching pairs of cards. Respondents could continue the task until they made all six matches, which nearly all did. Therefore, we analyzed the number of incorrect responses (lower score is better) made until reaching the end of the task. Matrix pattern completion assesses ability to correctly select an element missing in a visual pattern (higher score is better). Tower rearrangement assesses ability to plan a sequence of moves to rearrange elements of an image into a pre-specified arrangement (higher score is better). Symbol-digit substitution assesses psychomotor processing speed and executive function by the number of correct matches of symbols with single-digit integers within a time limit (higher score is better). Reaction time assesses mean time to correctly identify matching pairs of images (lower time is better). Trails: numeric assesses time to complete a numeric path by clicking sequentially on numbers scattered around a screen (lower time is better); Trails: alphanumeric assesses time to complete an alphanumeric path by clicking sequentially on alternating numbers and letters scattered around a screen (lower time is better). The UK Biobank provides further descriptions of each of these cognitive tasks at https://biobank.ctsu.ox.ac.uk/crystal/label.cgi?id=100026.

### Covariates

To control for potential confounding, we adjusted for variables that could potentially be associated with exposure to *T*. *gondii* and with cognitive function. We included covariates previously associated with brain structure and cognitive function [25] as well as others: age (years), sex (female, male), race-ethnicity (white, nonwhite), educational attainment (college degree, less than college degree), income (the midpoint of reported categories in 10,000 pounds/year: less than 18,000; 18,000 to 30,999; 31,000 to 51,999; 52,000 to 99,999; and 100,000£ and above), self-rated health (four-point scale ranging from poor to excellent), body-mass index (kg/m^2^), smoking history (non-smoker, past, current), and alcohol use (six categories ranging from never to daily or almost daily).

### Statistical analysis

We used Stata 16.1 (StataCorp, Stata Statistical Software, Release 16. College Station, Texas) for all statistical calculations. We estimated linear regression models to evaluate associations between each of four measures of *T*. *gondii* as the focal independent variable and measures of cognitive function as dependent variables. The four measures of *T*. *gondii* were *T*. *gondii* seropositivity, natural-log transformed p22 antibody level, natural-log transformed sag1 antibody level, and the mean of the natural-log transformed p22 and sag1 antibody levels after standardization. Because the measures of the cognitive tasks were continuous and met the assumption of normally distributed residuals, use of a linear regression model was appropriate.

We created five sets of linear regressions models for each focal predictor (i.e., *T*. *gondii* seropositivity, natural-log transformed p22 antibody level, natural-log transformed sag1 antibody level, and the mean of the natural-log transformed p22 and sag1 antibody levels). The first set of models estimated the relationship between each of the four focal predictors and the nine cognitive functioning outcomes, for a total of 36 models, each model adjusting for the preselected covariates. Interactions between each of the four focal predictors with age, sex, educational attainment, and income, again adjusting for the preselected covariates, make up the final four sets of regressions. Each *T*. *gondii* by predictor interaction consisted of 9 models, one for each measure of cognitive functioning. With four measures of *T*. *gondii* and four predictors to interact with, there are 144 interaction models for a total of 180 models.

With such a large number of hypothesis tests, our analyses face a notable risk of identifying false-positive relationships due to alpha inflation. Typical approaches to addressing the alpha inflation that results from large numbers of statistical tests, e.g., Bonferroni, adjust the p-value threshold to account for increased likelihood of rejecting hypotheses. Such approaches are often overly conservative, protecting against alpha inflation at the expense of statistical power [26, 27]. In the present case, we address the problem of multiple comparisons using the multivariate approach outlined by Rencher and Scott [26], who demonstrated with a simulation study that a multivariate test of the relationship between a single predictor (in this case, *T*. *gondii*) and multiple dependent variables preserves the traditional alpha level of .05, in part, by taking into account the joint covariance structure of the dependent variables. Traditionally, this multivariate approach is available using MANOVA procedures. In that context, conclusions of multivariate significance are determined based on the Wilks’ lambda, Hotelling-Lawley trace, Pillai’s trace, and Roy’s large root [27]. In the regression context, which we take in these analyses, we leverage Stata’s *suest* command [28] to estimate a multivariate test for each set of models. After estimating each individual model that belongs to a single set (i.e., one predictor or interaction and each of the 9 dependent variables), the *suest* command joins the models into a single parameter vector that includes the covariances of the dependent variables. Subsequently, instead of the traditional measures of multivariate significance, a single multivariate test calculated with the hypothesis that the relationship of the predictor and each of the outcomes is null. Therefore, if the probability that the multivariate null was above .05, we disregarded any significant univariate findings. In contrast, if the multivariate test was < .05, we considered the significant individual findings to be positive.

## Results

The average age of the final overall sample of 6,780 was 55.3 years, 55% were women, 95% were white, 41% had obtained a college degree, and 27% were *T*. *gondii* seropositive (Table 1). Table 1 also shows additional characteristics of the sample including additional demographics and average performance on the cognitive tasks.

**Fig 1:**
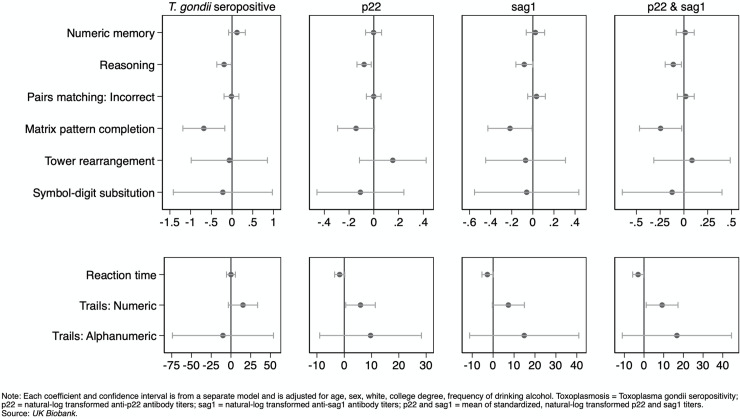
Cognitive Functioning and Seropositivity and Serointensity of *T*. *gondii*: Adjusted Coefficients and 95% Confidence Intervals from Linear Regression.

In adjusted models, *T*. *gondii* seropositivity was significantly associated with worse reasoning (b = -.192, *p* < .05) and worse matrix pattern completion (b = -.681, *p* < .01). The natural log of the p22 level was associated with worse reasoning (b = -.078, *p* < .01) and slower performance on Trails: numeric (b = 5.962, *p* < .05). The natural log of the sag1 level was associated with faster reaction time (b = -2.739, *p* < .05) but with worse reasoning (b = -.081, *p* < .05) and with worse matrix pattern completion (b = -.217, *p* < .05). The mean of the natural logs of the p22 and sag1 levels were significantly associated with faster reaction time (b = -3.000, *p* < .05) but with worse reasoning (b = -.112, *p* < .05), slower performance on Trails: numeric (b = 9.195, *p* < .05), and worse matrix pattern completion (-.245, *p* < 05). The multivariate tests were significant for *Toxoplasma gondii* seropositivity (*p* = .043), the natural log of the p22 level (*p* = .002), the natural log of the sag1 level (*p* = .018), and the mean of the natural log of the p22 and sag1 levels (*p* = .002) (Table 2, Fig 1).

**Table 2:** Adjusted models of cognitive functioning on *T*. *gondii*: Unstandardized coefficients from linear regression.

	*T*. *gondii* Seropositive	p22	sag1	Mean of p22 and sag1	N
Cognitive functioning					
Numeric memory	.119	-.002	.024	.013	795
Reasoning	-.192*	-.078**	-.081*	-.112*	2,267
Pairs matching: Incorrect	-.015	-.001	.034	.019	6,780
Matrix pattern completion	-.681**	-.143	-.217*	-.245*	312
Tower rearrangement	-.065	.153	-.069	.085	316
Symbol-digit substitution	-.222	-.108	-.059	-.124	313
Reaction time	.008	-1.673	-2.739*	-3.000*	6,752
Trails: Numeric	15.261	5.962*	7.293	9.195*	312
Trails: Alphanumeric	-10.121	9.645	14.860	16.699	301
Multivariate test^a^					
*p*	.043	.002	.018	.002	

Note: Each cell in the table represents the results from a separate model. The main independent variable is listed in the column headers and the dependent variable is listed in the row labels. Each model is adjusted for age, sex, white, college degree, household income, self-rated health, body-mass index, smoking status, and frequency of drinking alcohol.

^a^ The multivariate test is a test of the null hypothesis considered within the joint covariance of the dependent variables (i.e., cognitive functioning measures) that the measure of *T*. *gondii* (i.e., *T*. *gondii* seropositive, p22, sag1, combined p22 and sag1) is related to cognitive functioning. It is applied here to address potential problems of reporting false positives because of the number of statistical tests performed. Significant relationships between a *T*. *gondii* measure and cognitive function are thus ignored if the probability of the multivariate null being true is greater than .05. *T*. *gondii* = *Toxoplasma gondii* seropositivity; p22 = natural-log transformed anti-p22 antibody levels; sag1 = natural-log transformed anti-sag1 antibody levels; Mean of p22 and sag1 = mean of standardized, natural-log transformed p22 and sag1 levels.

* p < .05

** p < .01

*** p < .001. Source: *UK Biobank*.

**Table 3:** Multivariate *p*-values^a^ from adjusted models of the interactions of *T*. *gondii* with age, sex, education, and income.

	*T*. *gondii* seropositive	p22	sag1	Mean of p22 and sag1
Age x *T*. *gondii*	.137	.053	.779	.261
Female x *T*. *gondi*i	.166	.373	.004	.021
College degree x *T*. *gondii*	.653	.113	.158	.158
Income x *T*. *gondii*	.022	.005	.459	.054

Note

^a^ Each p-value represents a multivariate test, which is a test of the null hypothesis considered within the joint covariance of the dependent variables (i.e., all nine cognitive functioning measures) and the respective interaction between one of the *T*. *gondii* variables and a predictor (e.g., Age x *T*. *gondii* seropositive). Results of the models that are represented in these multivariate tests are presented in supplemental tables.

**Fig 2:**
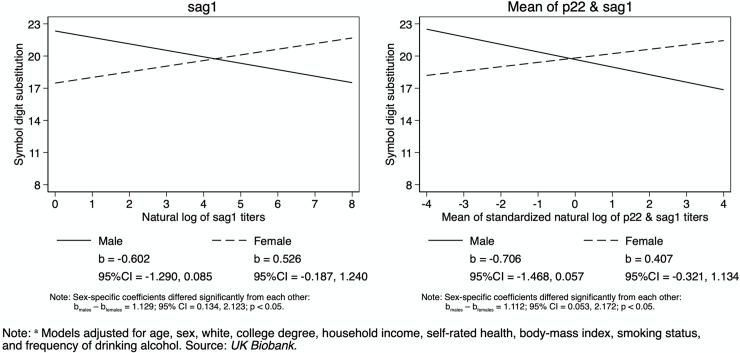
Interaction of *T*. *gondii* and Sex on Cognitive Functioning: Adjusted Predictions from Linear Regression.

**Fig 3:**
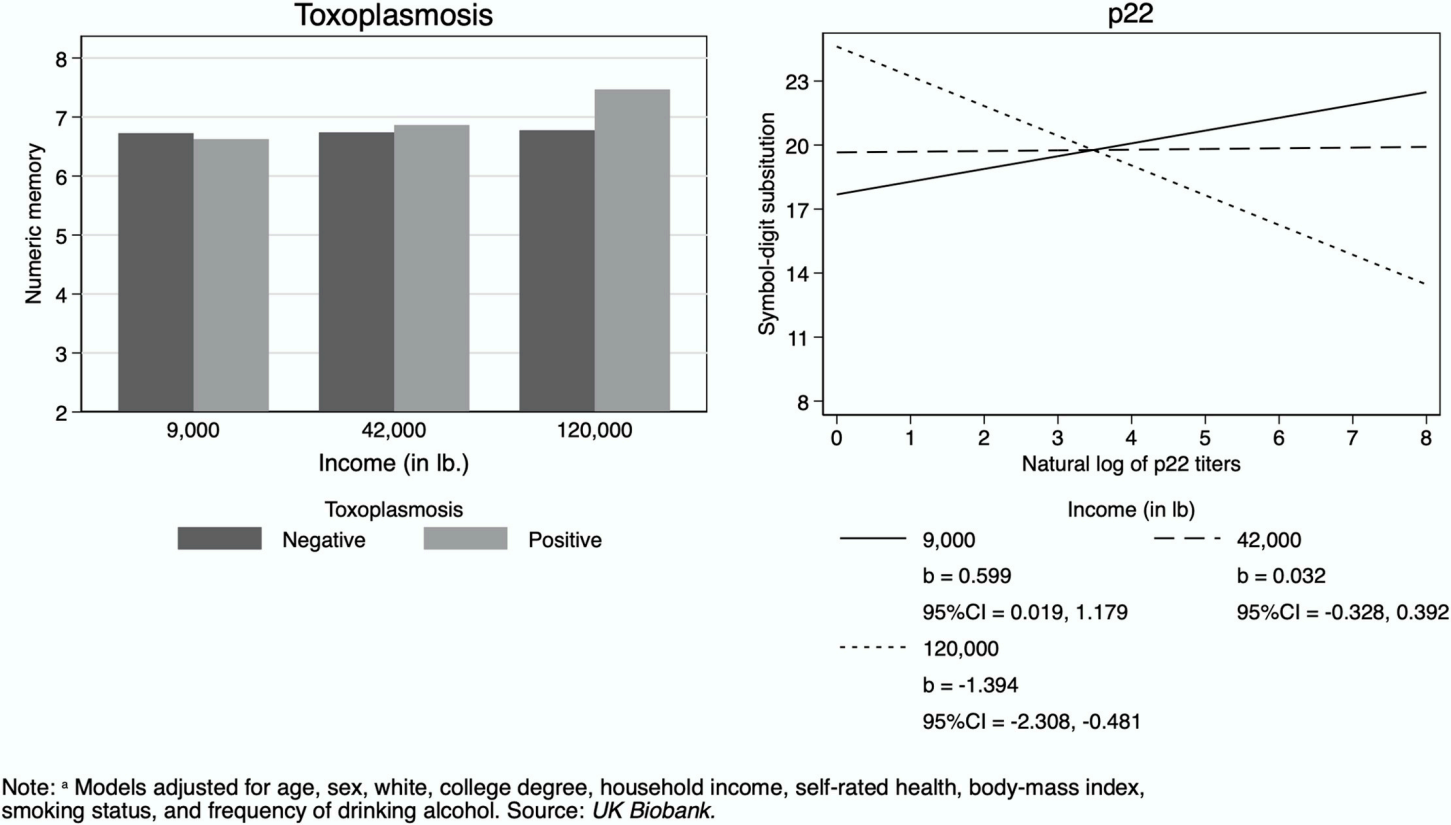
Interaction of *T*. *gondii* and Income on Cognitive Functioning: Adjusted Predictions from Linear Regression.

Multivariate tests of the relationship between cognitive functioning and interactions of *T*. *gondii* with age, sex, education, and income are reported in Table 3. Age did not significantly moderate any associations of *T*. *gondii* seropositivity or serointensity with cognitive function that withstood the multivariate test (S1 Table). Sex moderated the associations of the natural log of the sag1 level and the mean of the natural logs of p22 and sag1 levels in the multivariate test. Symbol-digit substitution was the cognitive measure responsible for the significant multivariate test (S2 Table). In men, higher serointensity was associated with lower symbol-digit substitution score, while in women, higher serointensity was associated with higher symbol-digit substitution score (Fig 2). Educational attainment did not significantly moderate any associations (S3 Table). The interaction of income and *T*. *gondii* seropositivity and the natural log of p22 were significant in their multivariate tests (S4 Table). *T*. *gondii* seropositivity was associated with similar or worse numeric memory at low levels of income, and with better numeric memory at middle to high levels of income (Fig 3). Income also moderated the association of the natural log of the p22 level with symbol-digit substitution. Serointensity was associated with similar or better digit-symbol substitution performance at low to medium levels of income, and with worse digit-symbol substitution performance at high income levels (Fig 3). Finally, we have included a supplemental figure (S1 Fig) in the Appendix consisting of the unadjusted bivariate relationships between *T*. *gondii* and each cognitive measure. This figure includes both violin plots and scatterplots.

## Discussion

In this community-based sample of adults in the United Kingdom, we analyzed associations of four measures of *Toxoplasma gondii* infection–seropositivity, natural-log transformed p22 antibody level, natural-log transformed sag1 antibody level, and mean of the natural-log transformed p22 and sag1antibody levels–and performance on nine cognitive tasks. In these analyses, the main findings are as follows: First, *Toxoplasma gondii* seropositivity was associated with worse reasoning and matrix pattern completion. Second, *Toxoplasma gondii* natural-log transformed p22 antibody levels were associated with worse reasoning and with worse performance on the Trails: numeric task. Third, *Toxoplasma gondii* natural-log transformed sag1 levels were associated with better reaction time but with worse reasoning and with worse matrix pattern completion. Fourth, the mean of the natural-log transformed p22 and sag1 levels was associated with better reaction time but with worse reasoning, worse performance on the Trails: numeric task, and worse matrix pattern completion. We did not find associations of *Toxoplasma gondii* seropositivity with any of the other measures of cognitive function.

Sample size requires consideration when interpreting these findings. The finding of an association between *Toxoplasma gondii* and worse reasoning came from a comparatively large sample size of 2,267. In contrast, the findings of an association between *Toxoplasma gondii* and worse performance with matrix pattern completion and on the Trails, numeric task came from smaller samples of 312. The association between *Toxoplasma gondii* and better reaction time came from a comparatively large sample of 6,752. The findings based on samples with comparatively small sample sizes possibly could be false positive findings due to small sample sizes, although these sample sizes were both still over 300. Further, the analyses showing no statistically significant associations between *Toxoplasma gondii* and cognitive function in the smaller samples could be underpowered.

While we found few interactive effects overall, sex moderated some associations between *Toxoplasma gondii* and symbol-digit substitution. However, the sex-specific associations were in opposite directions, and neither was itself significantly different from zero, even though the difference between sex-specific associations was significant. Income also moderated some associations between *Toxoplasma gondii* and numeric memory and symbol-digit substitution. In people with high income, *Toxoplasma gondii* seropositivity was associated with higher numeric memory score. However, higher natural-log transformed p22 levels were associated with worse symbol-digit substitution score in those with higher income.

Based on the available neuropsychological tasks, the associations we found between *Toxoplasma gondii* seropositivity and serointensity and cognitive function appeared to involve primarily executive function, not memory, although comparatively few tests of memory and even fewer tasks if any evaluating other cognitive domains such as language function and processing speed were available. Despite the adverse associations between *Toxoplasma gondii*, performance on the Trails: numeric task, and matrix pattern completion, we did not find associations with tower rearrangement, which is another task involving executive function. That is, in this dataset, *Toxoplasma gondii* infection was associated with some but not all tasks of executive function.

While these findings are consistent with findings from several previous studies indicating worse cognitive function associated with *Toxoplasma gondii* [12–16, 18, 22, 29], they differ from other published studies [19–21] that did not find associations between *Toxoplasma gondii* and cognitive function. Several possible factors could account for these different findings, including the use of different cognitive tasks. While Gale et al. [13] did not find main effects when evaluating associations between *Toxoplasma gondii* seropositivity and cognitive function, they did find interactions showing associations between *Toxoplasma gondii* seropositivity and symbol-digit substitution in groups with low education and income, similar to the findings in this study of income affecting the association between natural-log transformed sag1 level and the mean of the natural-log transformed p22 and sag1 levels and symbol-digit substitution, although we did not find in this study that educational attainment affected the association between *T*. *gondii* and symbol-digit substitution. Our findings also differ from those of Sugden et al. [20], who found no associations between *Toxoplasma gondii* seropositivity and cognitive function, except for lower performance on the Rey Auditory Verbal Learning test in the *Toxoplasma gondii* seropositive group. In contrast, we found no associations with memory except in the interaction models with numeric memory, finding instead evidence of lower executive function associated with *Toxoplasma gondii* seropositivity on some but not all tests of executive function, whereas Sugden et al. [20] found no associations between *Toxoplasma gondii* seropositivity and performance on the tasks they used to assess executive function.

In addition to the adverse associations between *Toxoplasma gondii* seropositivity and serointensity we found, we also observed associations between the natural-log transformed sag1 level and the mean of the natural-log transformed p22 and sag1 levels and better (faster) reaction time. While the association with faster reaction time is somewhat counterintuitive and in contrast to other studies [30], Stock et al., [31] reported associations between *Toxoplasma gondii* seropositivity and better cognitive function. In that study, *Toxoplasma gondii* seropositivity was associated with better action control, which the authors speculated might have been due to changes in dopamine transmission related to *Toxoplasma gondii* seropositivity [31]. Finally, one other study found some evidence of better performance in participants positive for *Toxoplasma gondii* on some but not all cognitive tests administered [32]. While unexpected, our finding of an association between *Toxoplasma gondii* serointensity and better reaction time comes from a sample size of 6,752, a large sample that minimizes the chance for error due to small sample. This finding suggests that not all cognitive effects associated with *Toxoplasma gondii* are necessarily adverse, although the better reaction time we found occurred within the context of worsened executive function. The associations between *Toxoplasma gondii* and dopamine synthesis and between dopamine and some cognitive functions [31] possibly could improve some aspect of cognitive function. It is feasible that the faster reaction time associated with *Toxoplasma gondii* we found could provide an evolutionary advantage offsetting some of the potential evolutionary disadvantage from lower executive function. Because humans are dead-end hosts for *Toxoplasma gondii*, another possibility is that there is no disadvantage to *Toxoplasma gondii* from any cognitive improvement in humans [31].

We found different associations with different markers of *T*. *gondii* (Table 2). That is, some markers of *T*. *gondii* were associated with performance on some cognitive tasks, whereas other markers were associated with performance on other cognitive tasks. While we did not design our study to identify the causes of these differences, we note that cut-off points for determining seropositivity and host factors including timing of infection, genetic, and immune status as well as different sensitivity and specificity profiles between the different antigens [33, 34] could account for some of these differences.

Whether the cognitive dysfunction associated with *Toxoplasma gondii* seropositivity in the UK Biobank sample is associated with risk for later dementia is unclear. However, cognitive function itself is associated with subsequent dementia [35], and a meta-analysis has found an association between *Toxoplasma gondii* seropositivity and dementia [22]. Together, the findings of associations between *Toxoplasma gondii* seropositivity and serointensity and cognitive function and dementia suggest that *Toxoplasma gondii* infection could be a novel risk factor for either all-cause dementia or specific types of dementia, although additional studies are required to further evaluate this hypothesis.

Although we did not design this study to investigate mechanisms by which *Toxoplasma gondii* infection could be associated cognitive function, several not necessarily exclusive mechanisms could account for the observed associations. *Toxoplasma gondii* can increase permeability across the gastrointestinal-blood border, potentially enabling entry of other pathogens or toxins into the systemic circulation and eventually across the blood-brain barrier [8]. *Toxoplasma gondii* also appears to affect several neurotransmitters including dopamine, gamma amino butyric acid, glutamate, and serotonin [36], actions that could be associated with cognitive function. In addition, *Toxoplasma gondii* might affect gene expression [37], and the *Toxoplasma gondii* cysts in the brain might alter brain function [38].

Our study has several strengths including objective exposure and outcome variables, inclusion of multiple covariates to control for potential confounding, use of interaction models to examine whether some groups might be more susceptible to cognitive effects from exposure to *Toxoplasma gondii*, and use of several measures of cognitive function including measures of executive function and memory. Our study also has several limitations that require consideration. While sample sizes for some of the cognitive tasks were large, some were smaller, possibly resulting in decreased statistical power to detect differences between *Toxoplasma gondii* seropositive and seronegative groups or increasing the chances of a type-one error. In addition, we do not have information about when the initial infection occurred or at which stage of development the initial infection occurred. It is possible that infection during childhood could have different effects on cognitive function from the effects of initial infection during adulthood, suggesting that the time of initial exposure could be a critical variable. Similarly, it is feasible that the stage of *Toxoplasma gondii* or the route of infection could affect associations with cognitive function. However, we did not have available data to statistically address these potentially confounding variables. In this regard, geographical place of birth and of residence variables could confound the association between *Toxoplasma gondii* and cognitive function. Unfortunately, we do not have access to current place of residence, and country of birth was the most fine-grained available variable for place of birth, which we felt was not specific enough because exposure to *Toxoplasma gondii* likely varies across communities. As such, we did not include geographical variables in the statistical models. While we adjusted for several variables that could lead to potential confounding, residual confounding still could affect our results. Finally, we used a cross-sectional study design, precluding causal determinations because we cannot determine whether exposure preceded cognitive changes.

In conclusion, in this community-based study of adults using data from the UK Biobank, adjusted models showed associations between *Toxoplasma gondii* and worse reasoning, worse matrix pattern completion, and worse performance on the Trails: numeric task, all of which assess executive function. In the context of potential limitations of this study, these finding are consistent with the results of several previous studies that have found associations between *Toxoplasma gondii* and lower cognitive function. The widespread distribution of *Toxoplasma gondii* and high prevalence of *Toxoplasma gondii* seropositivity and its potential adverse association with cognitive function indicate the need for additional studies characterizing the effects of *Toxoplasma gondii* on cognitive decline over time and the association between *Toxoplasma gondii* and risk for neurodegeneration.

## Supporting information

S1 Table Adjusted models of cognitive functioning on the interaction of *T*. *gondii* and age: Unstandardized coefficients from linear regression. (DOCX)

S2 Table Adjusted models of cognitive functioning on the interaction of *T*. *gondii* and sex: Unstandardized coefficients from linear regression. (DOCX)

S3 Table Adjusted models of cognitive functioning on the interaction of *T*. *gondii* and educational attainment: Unstandardized coefficients from linear regression. (DOCX)

S4 Table Adjusted models of cognitive functioning on the interaction of *T*. *gondii* and income (in 10,000 £.): Unstandardized coefficients from linear regression. (DOCX)

S1 Fig Bivariate relationships of *T*. *gondii* with cognition. (TIFF)
