# Supplementary material for: Toxoplasma gondii seropositivity and serointensity and cognitive function in adults
Source: PLoS Negl Trop Dis. 2020 Oct 15;14(10):e0008733. doi: 10.1371/journal.pntd.0008733 (PMC7561134; doi:10.1371/journal.pntd.0008733)
Supplement: S1 Table — (DOCX) [file pntd.0008733.s001.docx]

Supplemental Table 1.

Adjusted models of cognitive functioning on the interaction of *T. gondii* and age:

Unstandardized coefficients from linear regression

|  | | | | | |  |
| --- | --- | --- | --- | --- | --- | --- |
|  | *T. gondii*  seropositive | p22 | sag1 | Mean of  p22 and sag1 | N | |
| Numeric memory |  |  |  |  | 795 | |
| Toxo | .158 | .097 | -.015 | .065 |  | |
| Age | -.005 | .001 | -.008 | -.005 |  | |
| Toxo x Age | -.001 | -.002 | .001 | -.001 |  | |
| Reasoning |  |  |  |  | 2,267 | |
| Toxo | -.635 | .021 | -.033 | .005 |  | |
| Age | -.000 | .007 | .006 | .002 |  | |
| Toxo x Age | .008 | -.002 | -.001 | -.002 |  | |
| Pairs matching: Incorrect |  |  |  |  | 6,780 | |
| Toxo | -.244 | -.072 | -.068 | -.099 |  | |
| Age | .065*** | .061*** | .057* | .065*** |  | |
| Toxo x Age | .004 | .001 | .002 | .002 |  | |
| Matrix pattern completion |  |  |  |  | 312 | |
| Toxo | -4.340* | -1.325* | -1.404 | -2.077* |  | |
| Age | -.072*** | -.131*** | -.152* | -.054*** |  | |
| Toxo x Age | .067* | .022* | .022 | .034* |  | |
| Tower rearrangement |  |  |  |  | 316 | |
| Toxo | -3.566 | .769 | .512 | 1.064 |  | |
| Age | -.136*** | -.084 | -.075 | -.124*** |  | |
| Toxo x Age | .064 | -.011 | -.011 | -.018 |  | |
| Symbol digit substitution |  |  |  |  | 313 | |
| Toxo | -7.106 | -1.501 | .456 | -1.181 |  | |
| Age | -.255*** | -.311*** | -.184 | -.223*** |  | |
| Toxo x Age | .126 | .026 | -.009 | .020 |  | |
| Reaction time |  |  |  |  | 6,752 | |
| Toxo | 9.565 | 2.529 | 7.201 | 6.166 |  | |
| Age | 3.873*** | 4.104*** | 4.654*** | 3.856*** |  | |
| Toxo x Age | -.171 | -.076 | -.179 | -.165 |  | |
| Trails: Numeric |  |  |  |  | 312 | |
| Toxo | -47.024 | -6.550 | 21.890 | 8.557 |  | |
| Age | 2.304*** | 1.857 | 3.736 | 2.589*** |  | |
| Toxo x Age | 1.142 | .234 | -.270 | .012 |  | |
| Trails: Alphanumeric |  |  |  |  | 301 | |
| Toxo | 302.982 | -11.605 | 18.436 | 3.622 |  | |
| Age | 11.450*** | 8.789 | 10.240 | 10.045*** |  | |
| Toxo x Age | -5.788 | .399 | -.066 | .243 |  | |
| Multivariate test^a^ |  |  |  |  |  | |
| *p* | .137 | .053 | .779 | .261 |  | |
| Note: Each model is adjusted for age, sex, white, college degree, household income, self-rated health, body-mass index, smoking status, and frequency of drinking alcohol. ^a^ The multivariate test is a test of the null hypothesis considered within the joint covariance of the dependent variables (i.e., cognitive functioning measures) that age does not moderate the relationship between a measure of *T. gondii* (i.e., *T. gondii* seropositive, p22, sag1, combined p22 and sag1) and cognitive functioning. It is applied here to address potential problems of reporting false negatives because of the number of statistical tests performed. Significant interactions between a *T. gondii* measure and age are thus ignored if the probability of the multivariate null being true is greater than .05. *T. gondii* = Toxoplasma gondii seropositivity; p22 = natural-log transformed anti-p22 antibody levels; sag1 = natural-log transformed anti-sag1 antibody levels; Mean of p22 and sag1 = mean of standardized, natural-log transformed p22 and sag1 levels.  * p < .05, ** p < .01, *** p < .001. Source: *UK Biobank*. | | | | | |  |
