# Supplementary material for: Toxoplasma gondii seropositivity and serointensity and cognitive function in adults
Source: PLoS Negl Trop Dis. 2020 Oct 15;14(10):e0008733. doi: 10.1371/journal.pntd.0008733 (PMC7561134; doi:10.1371/journal.pntd.0008733)
Supplement: S2 Table — (DOCX) [file pntd.0008733.s002.docx]

Supplemental Table 2.

Adjusted models of cognitive functioning on the interaction of *T. gondii* and sex:

Unstandardized coefficients from linear regression

|  | | | | | |
| --- | --- | --- | --- | --- | --- |
|  | *T. gondii*  seropositive | p22 | sag1 | Mean of  p22 and sag1 | N |
| Numeric memory |  |  |  |  | 795 |
| Toxo | .034 | -.024 | -.025 | -.035 |  |
| Female | -.460*** | -.552* | -.769 | -.409*** |  |
| Toxo x Female | .158 | .041 | .081 | .083 |  |
| Reasoning |  |  |  |  | 2,267 |
| Toxo | -.052 | -.018 | -.047 | -.042 |  |
| Female | -.162 | .150 | .047 | -.238** |  |
| Toxo x Female | -.262 | -.115* | -.063 | -.130 |  |
| Pairs matching: Incorrect |  |  |  |  | 6,780 |
| Toxo | -.036 | -.012 | -.011 | -.016 |  |
| Female | .002 | -.056 | -.345 | .014 |  |
| Toxo x Female | .040 | .020 | .081 | .065 |  |
| Matrix pattern completion |  |  |  |  | 312 |
| Toxo | -.871* | -.181 | -.329* | -.338* |  |
| Female | -.658** | -.771 | -1.517 | -.517* |  |
| Toxo x Female | .384 | .063 | .233 | .176 |  |
| Tower rearrangement |  |  |  |  | 316 |
| Toxo | -.203 | -.057 | -.150 | -.138 |  |
| Female | -1.061* | -2.117* | -1.695 | -.930* |  |
| Toxo x Female | .276 | .353 | .165 | .422 |  |
| Symbol digit substitution |  |  |  |  | 313 |
| Toxo | -.373 | -.451 | -.602 | -.706 |  |
| Female | -.111 | -1.924 | -4.860* | .138 |  |
| Toxo x Female | .302 | .577 | 1.129* | 1.112* |  |
| Reaction time |  |  |  |  | 6,752 |
| Toxo | 1.430 | -1.060 | -3.758 | -3.069 |  |
| Female | 18.205*** | 21.095** | 9.505 | 17.375*** |  |
| Toxo x Female | -2.643 | -1.111 | 1.802 | .124 |  |
| Trails: Numeric |  |  |  |  | 312 |
| Toxo | 24.154 | 6.511 | 3.658 | 6.889 |  |
| Female | .219 | -.310 | -37.460 | -3.599 |  |
| Toxo x Female | -17.825 | -.925 | 7.561 | 4.408 |  |
| Trails: Alphanumeric |  |  |  |  | 301 |
| Toxo | -25.323 | 7.309 | 3.306 | 7.105 |  |
| Female | 19.280 | 15.116 | -78.686 | 29.244 |  |
| Toxo x Female | 31.079 | 3.926 | 24.116 | 18.335 |  |
| Multivariate test |  |  |  |  |  |
| *p* | .166 | .373 | .004 | .021 |  |
| Note: Each model is adjusted for age, sex, white, college degree, household income, self-rated health, body-mass index, smoking status, and frequency of drinking alcohol. ^a^ The multivariate test is a test of the null hypothesis considered within the joint covariance of the dependent variables (i.e., cognitive functioning measures) that sex does not moderate the relationship between a measure of *T. gondii* (i.e., *T. gondii* seropositive, p22, sag1, combined p22 and sag1) and cognitive functioning. It is applied here to address potential problems of reporting false negatives because of the number of statistical tests performed. Significant interactions between a *T. gondii* measure and sex are thus ignored if the probability figure of the multivariate null being true is greater than .05. *T. gondii* = Toxoplasma gondii seropositivity; p22 = natural-log transformed anti-p22 antibody levels; sag1 = natural-log transformed anti-sag1 antibody levels ; Mean of p22 and sag1 = mean of standardized, natural-log transformed p22 and sag1 levels. * p < .05, ** p < .01, *** p < .001. Source: *UK Biobank*. | | | | | |
