# Supplementary material for: Toxoplasma gondii seropositivity and serointensity and cognitive function in adults
Source: PLoS Negl Trop Dis. 2020 Oct 15;14(10):e0008733. doi: 10.1371/journal.pntd.0008733 (PMC7561134; doi:10.1371/journal.pntd.0008733)
Supplement: S3 Table — (DOCX) [file pntd.0008733.s003.docx]

Supplemental Table 3.

Adjusted models of cognitive functioning on the interaction of *T. gondii* and educational attainment: Unstandardized coefficients from linear regression

|  | | | | | |  |
| --- | --- | --- | --- | --- | --- | --- |
|  | *T. gondii*  seropositive | p22 | sag1 | Mean of  p22 and sag1 | N | |
| Numeric memory |  |  |  |  | 795 | |
| Toxo | .113 | -.016 | .019 | -.001 |  | |
| College degree | .276* | .155 | .225 | .282** |  | |
| Toxo x College degree | .016 | .037 | .013 | .037 |  | |
| Reasoning |  |  |  |  | 2,267 | |
| Toxo | -.047 | -.055 | -.014 | -.053 |  | |
| College degree | 1.065*** | 1.179*** | 1.700*** | .971*** |  | |
| Toxo x College degree | -.351 | -.060 | -.165* | -.145 |  | |
| Pairs matching: Incorrect |  |  |  |  | 6,780 | |
| Toxo | -.013 | -.034 | .050 | .002 |  | |
| College degree | -.103 | -.368 | .066 | -.103 |  | |
| Toxo x College degree | -.003 | .078 | -.038 | .041 |  | |
| Matrix pattern completion |  |  |  |  | 312 | |
| Toxo | -.626 | -.132 | -.235 | -.247 |  | |
| College degree | .165 | .251 | .018 | .161 |  | |
| Toxo x College degree | -.112 | -.021 | .032 | .002 |  | |
| Tower rearrangement |  |  |  |  | 316 | |
| Toxo | .161 | .070 | -.185 | -.047 |  | |
| College degree | .022 | -.554 | -.977 | -.035 |  | |
| Toxo x College degree | -.473 | .154 | .203 | .236 |  | |
| Symbol digit substitution |  |  |  |  | 313 | |
| Toxo | -.027 | .012 | .224 | .138 |  | |
| College degree | .735 | 1.371 | 2.822 | .583 |  | |
| Toxo x College degree | -.408 | -.224 | -.502 | -.473 |  | |
| Reaction time |  |  |  |  | 6,752 | |
| Toxo | -1.431 | -2.657* | -4.173* | -4.686* |  | |
| College degree | -4.782 | -11.742 | -19.091 | -3.741 |  | |
| Toxo x College degree | 3.537 | 2.345 | 3.442 | 3.998 |  | |
| Trails: Numeric |  |  |  |  | 312 | |
| Toxo | 17.935 | 3.882 | 5.341 | 6.373 |  | |
| College degree | -4.300 | -18.574 | -20.418 | -4.757 |  | |
| Toxo x College degree | -5.605 | 3.862 | 3.465 | 5.097 |  | |
| Trails: Alphanumeric |  |  |  |  | 301 | |
| Toxo | -14.755 | 15.171 | -4.927 | 10.019 |  | |
| College degree | -20.716 | 16.037 | -164.257 | -13.052 |  | |
| Toxo x College degree | 9.509 | -10.002 | 34.767 | 11.836 |  | |
| Multivariate test |  |  |  |  |  | |
| *p* | .653 | .113 | .158 | .158 |  | |
| Note: Each model is adjusted for age, sex, white, college degree, household income, self-rated health, body-mass index, smoking status, and frequency of drinking alcohol. ^a^ The multivariate test is a test of the null hypothesis considered within the joint covariance of the dependent variables (i.e., cognitive functioning measures) that educational attainment does not moderate the relationship between a measure of *T. gondii* (i.e., *T. gondii* seropositive, p22, sag1, combined p22 and sag1) and cognitive functioning. It is applied here to address potential problems of reporting false negatives because of the number of statistical tests performed. Significant interactions between a *T. gondii* measure and educational attainment are thus ignored if the probability of the multivariate null being true is greater than .05. *T. gondii* = Toxoplasma gondii seropositivity; p22 = natural-log transformed anti-p22 antibody levels; sag1 = natural-log transformed anti-sag1 antibody levels; Mean of p22 and sag1 = mean of standardized, natural-log transformed p22 and sag1 levels. * p < .05, ** p < .01, *** p < .001. Source: *UK Biobank*. | | | | | |  |
