# Supplementary material for: Toxoplasma gondii seropositivity and serointensity and cognitive function in adults
Source: PLoS Negl Trop Dis. 2020 Oct 15;14(10):e0008733. doi: 10.1371/journal.pntd.0008733 (PMC7561134; doi:10.1371/journal.pntd.0008733)
Supplement: S4 Table — (DOCX) [file pntd.0008733.s004.docx]

Supplemental Table 4.

Adjusted models of cognitive functioning on the interaction of *T. gondii* and income

(in 10,000 £.): Unstandardized coefficients from linear regression

|  | | | | | |
| --- | --- | --- | --- | --- | --- |
|  | *T. gondii*  seropositive | p22 | sag1 | Mean of  p22 and sag1 | N |
| Numeric memory |  |  |  |  | 795 |
| Toxo | -.162 | -.027 | -.049 | -.054 |  |
| Income | .004 | .001 | -.050 | .023 |  |
| Toxo x Income | .068* | .006 | .017 | .016 |  |
| Reasoning |  |  |  |  | 2,267 |
| Toxo | -.253 | -.115* | -.074 | -.137 |  |
| Income | .084*** | .060 | .095 | .088*** |  |
| Toxo x Income | .014 | .008 | -.002 | .006 |  |
| Pairs matching: Incorrect |  |  |  |  | 6,780 |
| Toxo | -.134 | -.070 | -.001 | -.058 |  |
| Income | -.019 | -.065 | -.047 | -.012 |  |
| Toxo x Income | .027 | .015 | .008 | .018 |  |
| Matrix pattern completion |  |  |  |  | 312 |
| Toxo | -.841 | -.007 | -.120 | -.061 |  |
| Income | .001 | .095 | .088 | .001 |  |
| Toxo x Income | .032 | -.027 | -.019 | -.038 |  |
| Tower rearrangement |  |  |  |  | 316 |
| Toxo | 1.218 | .369 | .267 | .459 |  |
| Income | .092 | .175 | .319 | .024 |  |
| Toxo x Income | -.254 | -.043 | -.067 | -.077 |  |
| Symbol digit substitution |  |  |  |  | 313 |
| Toxo | 1.010 | .754* | .520 | .898 |  |
| Income | .086 | .598** | .525 | .006 |  |
| Toxo x Income | -.243 | -.172** | -.115 | -.211* |  |
| Reaction time |  |  |  |  | 6,752 |
| Toxo | 5.904 | -2.001 | -2.122 | -2.890 |  |
| Income | -1.420** | -2.032 | -1.163 | -1.782*** |  |
| Toxo x Income | -1.345 | .073 | -.140 | -.025 |  |
| Trails: Numeric |  |  |  |  | 312 |
| Toxo | 13.030 | 6.709 | 6.528 | 8.458 |  |
| Income | -.759 | -.062 | -1.240 | -.562 |  |
| Toxo x Income | .441 | -.149 | .152 | .152 |  |
| Trails: Alphanumeric |  |  |  |  | 301 |
| Toxo | 8.504 | 9.496 | 10.173 | 12.073 |  |
| Income | -2.095 | -3.170 | -7.085 | -2.997 |  |
| Toxo x Income | -3.599 | .029 | .924 | .939 |  |
| Multivariate test |  |  |  |  |  |
| *p* | .022 | .005 | .459 | .054 |  |
| Note: Each model is adjusted for age, sex, white, college degree, household income, self-rated health, body-mass index, smoking status, and frequency of drinking alcohol. ^a^ The multivariate test is a test of the null hypothesis considered within the joint covariance of the dependent variables (i.e., cognitive functioning measures) that income does not moderate the relationship between a measure of *T. gondii* (i.e., *T. gondii* seropositive, p22, sag1, combined p22 and sag1) and cognitive functioning. It is applied here to address potential problems of reporting false negatives because of the number of statistical tests performed. Significant interactions between a *T. gondii* measure and income are thus ignored if the probability of the multivariate null being true is greater than .05. *T. gondii* = Toxoplasma gondii seropositivity; p22 = natural-log transformed anti-p22 antibody levels; sag1 = natural-log transformed anti-sag1 antibody levels; Mean of p22 and sag1 = mean of standardized, natural-log transformed p22 and sag1 levels. * p < .05, ** p < .01, *** p < .001. Source: *UK Biobank*. | | | | | |
