# Supplementary figures and images for: Toxoplasma gondii seropositivity and serointensity and cognitive function in adults
Source: PLoS Negl Trop Dis. 2020 Oct 15;14(10):e0008733. doi: 10.1371/journal.pntd.0008733 (PMC7561134; doi:10.1371/journal.pntd.0008733)

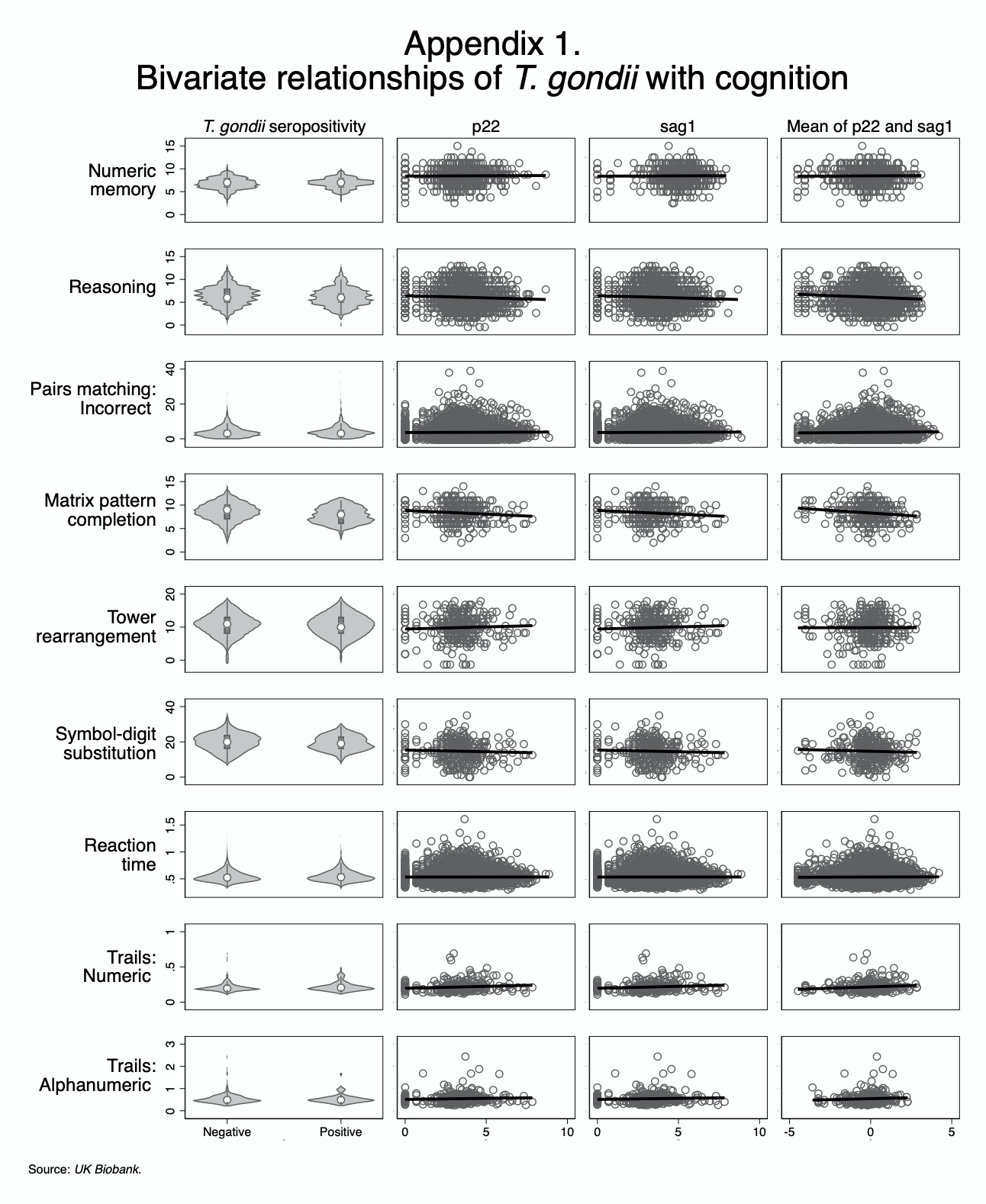

Supplement: S1 Fig — (TIFF) [file pntd.0008733.s005.tiff]
